# A Case of Strangulated Intestinal Obstruction Caused by Acquired Ileal Diverticulum

**DOI:** 10.1155/crgm/5634075

**Published:** 2025-06-12

**Authors:** Zhong-Yu Wang, Zhe Han, Hong-Fei Pang, Yu-Hang Liu, Ming Wei, Yuan-Yuan Wang

**Affiliations:** Gastrointestinal Disease Center, The First Hospital of Hebei Medical University, Shijiazhuang, Hebei, China

**Keywords:** acquired ileal diverticulum, case report, internal hernia, strangulated intestinal obstruction

## Abstract

**Background:** Acquired ileal diverticulum is an extremely rare condition that occurs in the ileum and is caused by acquired factors. Strangulated intestinal obstruction, a life-threatening variant of bowel obstruction, is associated with exceedingly high mortality rates. Here, we present a case of acquired ileal diverticulum causing strangulated intestinal obstruction, which was treated at our hospital.

**Case Report:** A 65-year-old female with no previous history of intestinal obstruction presented with acute abdominal pain. An exploratory laparotomy revealed an acquired ileal diverticulum and an internal hernia.

**Conclusion:** Acquired ileal diverticulum leading to strangulated intestinal obstruction is rare. Clinicians should consider the possibility of this disease when encountering intestinal obstruction patients with no history of abdominal surgery.

## 1. Introduction

Small intestinal diverticulum, a rare clinical entity classified by pathogenesis into congenital and acquired variants, demonstrates Meckel's diverticulum as the predominant congenital form while acquired types predominantly localize to the duodenum, jejunum, and ileum [1, 2]. The reported prevalence of acquired diverticulum in the small intestine ranges from 0.1% to 1.4% [3]. The current literature predominantly focuses on congenital obstruction resulting from embryological anomalies in Meckel's diverticulum, thus there remains a paucity of reports addressing the unique “intestinal wall degeneration-pedicle formation” mechanism specific to acquired ileal diverticula that leads to strangulated intestinal obstruction. We herein delineate a novel case of strangulated intestinal obstruction arising from an acquired ileal diverticulum, manifesting with acute abdomen as the primary clinical presentation.

## 2. Case Presentation

A 65-year-old female presented to our gastroenterology department with a two-day history of abdominal pain, bloating, and vomiting. The symptoms began suddenly, and she had no prior surgical history.

**Figure 1:**
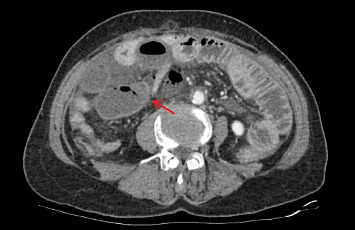
The obstruction site.

On abdominal examination, tenderness was noted throughout the abdomen, accompanied by rebound tenderness and muscle rigidity. Laboratory tests revealed an elevated white blood cell (WBC) count of 27.3 × 10^9^/L, with a neutrophil percentage of 92.6%, and an increased C-reactive protein (CRP) level of 150.01 mg/L. The patient's blood glucose was elevated at 7.24 mmol/L, and procalcitonin (PCT) was raised at 2.14 ng/mL. Other test results were unremarkable. The contrast-enhanced abdominal CT scan revealed dilated small bowel with fluid accumulation in the lower abdomen, demonstrating multiple air-fluid levels. Reduced enhancement of the small bowel wall was observed in the right lower quadrant. These findings are suggestive of strangulated small bowel obstruction, with suspicion of internal hernia formation (Figure 1).

**Figure 2:**
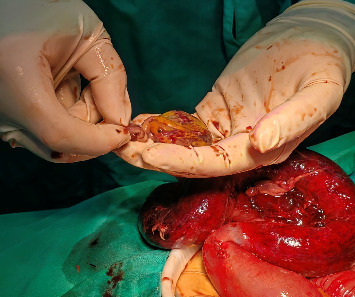
An ileal diverticulum with a 5-cm-long diverticular pedicle.

**Figure 3:**
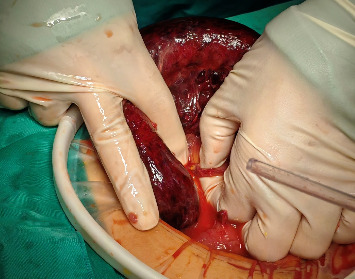
Hernia of the small intestine.

**Figure 4:**
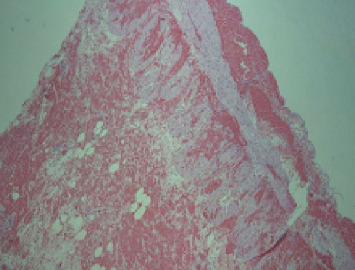
Pathology of the resected small intestine.

**Figure 5:**
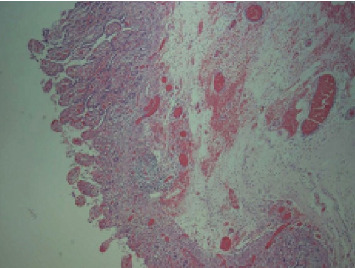
Pathology of the ileal diverticulum.

Considering the patient's medical history, physical examination findings, and supplementary diagnostic tests, a suspicion of strangulated intestinal obstruction with diffuse peritonitis was raised, indicating the need for surgical intervention. Therefore, an emergency surgery was performed. Following successful general anesthesia, the patient was positioned supine. The surgical area was sterilized and draped. A 1-cm curvilinear incision was made at the superior umbilical margin. After establishing carbon dioxide pneumoperitoneum, a 10-mm trocar was inserted for laparoscopic exploration. The procedure revealed distended and cyanotic segments of small bowel. Given the concern that laparoscopic management might delay definitive treatment, the decision was made to convert to open laparotomy. A right paramedian incision approximately 12 cm in length was created, and the abdominal cavity was entered layer by layer. After a thorough examination of the small intestine, the cause of the obstruction was identified. An ileal diverticulum, measuring approximately 3 × 3 × 4 cm and located about 40 cm from the ileocecal valve, was found. It had a 5-cm-long diverticular pedicle entangling part of the ileum, causing an internal hernia (Figures 2 and 3). Once the obstruction was cleared, a hot compress with wet gauze was applied to the intestine and its mesentery. However, there was no improvement in the color of the intestine, and the mesenteric blood vessels failed to regain pulsation, indicating intestinal necrosis. An intestinal resection was performed on the ischemic ileal segment containing the diverticulum, followed by a side-to-side anastomosis. A drainage tube was placed in the Douglas pouch, and another was positioned near the anastomosis. The resected specimen underwent histopathological examination, revealing acute and chronic inflammation of the intestinal mucosa in the approximately 30-cm-long resected segment (Figure 4), with areas of full-thickness necrosis and mesenteric tissue necrosis and hemorrhage, accompanied by acute inflammation. The ileal diverticulum measured approximately 6.0 × 5.0 × 1.5 cm (Figure 5), with full-thickness necrosis of the diverticular wall, acute and chronic inflammation, inflammatory exudation on the serosal surface, and no muscularis propria observed, confirming the lesion as an ileal false diverticulum.

Postoperatively, the patient was transferred to the intensive care unit (ICU) and started on antibiotics. On the first day after surgery, she was successfully moved to the general ward, where she received anti-inflammatory treatment, fluid resuscitation, regular dressing changes, and timely removal of the abdominal drainage tube. On postoperative Day 4, the patient's laboratory parameters demonstrated favorable progression: the WBC count normalized to 7.0 × 10^9^/L, accompanied by a decline in neutrophil percentage to 75.3% and a significant reduction in PCT to 0.38 ng/mL. The patient's CRP decreased to 28.79 mg/L. Her condition improved significantly, and she was discharged in stable condition on the 10th postoperative day.

## 3. Discussion

The exact cause of acquired ileal diverticulum remains unclear but may be linked to developmental abnormalities, increased intraluminal pressure, or adhesion and traction from surrounding tissues. Similar to Meckel diverticulum, acquired ileal diverticula also predominantly occur in the terminal ileum. Gross morphological differentiation between these two entities is challenging, necessitating histological verification. The congenital ileal diverticulum (Meckel diverticulum) characteristically demonstrates the four-layer mural architecture of the intestinal wall, whereas acquired diverticula exhibit mucosal herniation through the muscularis propria, lacking complete muscular layer integrity. Histopathological examination of the resected intestinal segment and diverticulum in this patient revealed full-thickness necrosis of the resected segment, with no muscularis propria present in the diverticulum. We believe the formation of the diverticulum in this patient is due to the absence of the muscular layer in the intestinal wall. Given that the diverticulum was located just 40 cm from the ileocecal valve, the patient was ultimately diagnosed with an acquired ileal diverticulum complicated by strangulated intestinal obstruction. This type of diverticulum is more common in adults and tends to increase with age [4]. Due to the unique blind pouch-like structure of the ileal diverticulum, intestinal contents can enter and accumulate within it through the diverticular neck. This accumulation may lead to complications such as diverticulitis, diverticular bleeding, or even perforation. If the contents of the diverticulum are extensive or the diverticular pedicle is long, it may compress or entangle surrounding bowel loops, resulting in complications such as internal hernia and intestinal obstruction. Intestinal obstruction accounts for approximately 20% of emergency abdominal surgeries [5] and can be classified into simple and strangulated types based on the presence of blood flow impairment in the intestinal segment. Strangulated intestinal obstruction can occur as a complication of simple intestinal obstruction due to the increased accumulation of intestinal contents, leading to bowel dilation. This results in ischemia and necrosis of the intestinal wall. It can also develop when the bowel becomes trapped in a hernia ring, causing similar consequences. Both conditions can lead to the leakage of toxins from the intestinal lumen and the migration of bacteria into the abdominal cavity, resulting in peritonitis and potentially septic shock. Strangulated intestinal obstruction carries a significantly higher mortality rate, estimated to be 2 to 10 times greater than that of nonstrangulated intestinal obstruction [6]. Therefore, surgical intervention should be promptly performed once strangulated intestinal obstruction is diagnosed.

Regarding the treatment of diverticula, patients with acquired ileal diverticulum do not require intervention unless clinical symptoms are present [7]. Conservative treatment, including fasting, anti-inflammatory therapy, and fluid resuscitation, should be considered for diverticulitis. However, if this approach fails or if severe complications such as bleeding, obstruction, or perforation occur, immediate surgical resection is required.

Strangulated intestinal obstruction caused by acquired ileal diverticulum is rare, making early diagnosis and intervention crucial. Surgeons should thoroughly evaluate obstructive symptoms, conduct a comprehensive physical examination, and carefully analyze relevant imaging studies. If imaging indicates a strangulated obstruction due to an internal hernia, acquired ileal diverticulum should be considered as a potential cause. Prompt surgical exploration is necessary to identify the source, minimize toxin absorption, and prevent septic shock.

## 4. Conclusions

Acquired ileal diverticulum is a rare condition that can result in both acute and chronic gastrointestinal issues. In cases of acute mechanical intestinal obstruction, clinicians should include acquired ileal diverticulum in the differential diagnosis. Surgical intervention is recommended for patients experiencing strangulated intestinal obstruction due to acquired ileal diverticulum.

## Data Availability

The data that support the findings of this study are available from the corresponding author upon reasonable request.
